# An Explainable Approach to Parkinson’s Diagnosis Using the Contrastive Explanation Method—CEM

**DOI:** 10.3390/diagnostics15162069

**Published:** 2025-08-18

**Authors:** Ipek Balikci Cicek, Zeynep Kucukakcali, Birgul Deniz, Fatma Ebru Algül

**Affiliations:** 1 Department of Biostatistics and Medical Informatics, Faculty of Medicine, Inonu University, 44280 Malatya, Turkey; zeynep.tunc@inonu.edu.tr; 2 Department of Hematology, Faculty of Medicine, Inonu University, 44280 Malatya, Turkey; birgul.deniz@inonu.edu.tr; 3 Department of Neurology, Faculty of Medicine, Inonu University, 44280 Malatya, Turkey; ebruycl86@yahoo.com

**Keywords:** Parkinson’s disease, explainable artificial intelligence, contrastive explanation method (CEM), deep learning, neuromorphological biomarkers

## Abstract

**Background/Objectives:** Parkinson’s disease (PD) is a progressive neurodegenerative disorder that requires early and accurate diagnosis. This study aimed to classify individuals with and without PD using volumetric brain MRI data and to improve model interpretability using explainable artificial intelligence (XAI) techniques. **Methods:** This retrospective study included 79 participants (39 PD patients, 40 controls) recruited at Inonu University Turgut Ozal Medical Center between 2013 and 2025. A deep neural network (DNN) was developed using a multilayer perceptron architecture with six hidden layers and ReLU activation functions. Seventeen volumetric brain features were used as the input. To ensure robust evaluation and prevent overfitting, a stratified five-fold cross-validation was applied, maintaining class balance in each fold. Model transparency was explored using two complementary XAI techniques: the Contrastive Explanation Method (CEM) and Local Interpretable Model-Agnostic Explanations (LIME). CEM highlights features that support or could alter the current classification, while LIME provides instance-based feature attributions. **Results:** The DNN model achieved high diagnostic performance with 94.1% accuracy, 98.3% specificity, 90.2% sensitivity, and an AUC of 0.97. The CEM analysis suggested that reduced hippocampal volume was a key contributor to PD classification (–0.156 PP), whereas higher volumes in the brainstem and hippocampus were associated with the control class (+0.035 and +0.150 PP, respectively). The LIME results aligned with these findings, revealing consistent feature importance (mean = 0.1945) and faithfulness (0.0269). Comparative analyses showed different volumetric patterns between groups and confirmed the DNN’s superiority over conventional machine learning models such as SVM, logistic regression, KNN, and AdaBoost. **Conclusions:** This study demonstrates that a deep learning model, enhanced with CEM and LIME, can provide both high diagnostic accuracy and interpretable insights for PD classification, supporting the integration of explainable AI in clinical neuroimaging.

## 1. Introduction

Parkinson’s disease (PD) is a progressive neurodegenerative disorder marked by the loss of dopaminergic neurons in the substantia nigra, resulting in a spectrum of motor and non-motor symptoms, including bradykinesia, rigidity, resting tremor, postural instability, cognitive impairment, depression, and sleep disturbances [1,2,3]. A key pathological feature of PD is the accumulation of alpha-synuclein aggregates, known as Lewy bodies, which lead to widespread neuronal degeneration and structural brain changes over time [1,4].

Neuroimaging studies have revealed that PD-related neurodegeneration is often accompanied by measurable volumetric alterations, particularly in subcortical and limbic regions. Structural MRI findings show reductions in areas such as the putamen, thalamus, and amygdala, which have been associated with motor severity and cognitive dysfunction [5,6,7]. Diffusion tensor imaging further demonstrates white matter disruptions and altered connectivity patterns, possibly indicating glymphatic system dysfunction and impaired network integration [8,9,10]. These findings highlight the potential of brain volumetric biomarkers in early diagnosis and disease monitoring.

Parallel to advances in imaging, artificial intelligence (AI) has become increasingly prominent in PD research, with machine learning (ML) and deep learning (DL) techniques being widely used for classification and prognosis. Algorithms such as support vector machines (SVMs), convolutional neural networks (CNNs), and ensemble models have achieved a high accuracy in distinguishing PD patients from healthy individuals using structural and functional neuroimaging data [10,11]. However, the lack of interpretability in these black box models remains a significant limitation, particularly in clinical settings where understanding the rationale behind diagnostic decisions is crucial.

To address this challenge, explainable artificial intelligence (XAI) approaches have gained substantial attention in recent PD studies. SHAP and LIME are among the most widely adopted methods, offering local explanations by estimating feature contributions around individual predictions. Several studies have demonstrated the utility of these tools across diverse data modalities. For example, SHAP has been used in ensemble models to interpret voice signal features for early PD detection [12], and in multimodal settings to explore disease progression predictors [13,14]. Other works have integrated SHAP with XGBoost or SVM models to analyze clinical or imaging biomarkers for diagnosing PD or predicting cognitive decline [15,16,17]. Furthermore, LIME and SHAP have been applied to benchmark classical ML models such as decision trees, logistic regression, and boosting algorithms, both for disease classification [18] and severity assessment [19]. In addition, multimodal time series data have been leveraged to train LightGBM, random forest, and SVM models, with enhanced explainability achieved through the combined use of SHAP and LIME frameworks [20].

Although attribution-based methods such as SHAP and LIME have enhanced model interpretability, they primarily highlight feature importance but do not consider counterfactual reasoning, that is, identifying the minimal changes necessary to alter a prediction. This type of reasoning is essential for effectively replicating clinical decision-making processes.

To fill this methodological gap, the present study integrates the Contrastive Explanation Method (CEM) into a deep neural network (DNN) framework to classify individuals with and without PD based on volumetric measurements from structural brain MRI. Unlike traditional attribution methods, CEM provides contrastive, individualized explanations by identifying both Pertinent Positives (features essential for maintaining a diagnosis) and Pertinent Negatives (features whose presence would change the outcome). This dual-layered approach mirrors the way clinicians reason through both confirming and excluding diagnostic possibilities.

Accordingly, the present study aims to classify PD using a deep neural network trained on volumetric brain features, while enhancing interpretability via CEM-based explanations. In contrast to previous work that relied on opaque predictive models, our approach delivers accurate classification alongside clinically relevant, patient-specific insights. This contributes to building more transparent, trustworthy, and actionable AI tools in the context of neurodegenerative disease diagnosis.

To facilitate the understanding of the study’s structure, the remainder of the manuscript is organized as follows: Section 2 describes the dataset, volumetric brain features, the detailed architecture of the deep neural network model, the training procedure, and the implementation of the CEM, along with the evaluation of time-based computational performance metrics for each processing unit. Section 3 presents the experimental results, including both model performance metrics and the interpretability analyses conducted using CEM. In addition, a statistical validation of the model-derived findings is included to strengthen the biological plausibility of the interpretability results. This section also incorporates a comparative table summarizing relevant studies from the literature. Section 4 discusses the clinical implications of the findings and contextualizes them within the broader field of neurodegenerative disease research. Finally, Section 5 concludes the paper by highlighting the key findings, underscoring the importance of explainable artificial intelligence in medical diagnostics, and offering directions for future research.

## 2. Materials and Methods

This section outlines the methodology adopted in the development of an explainable deep learning model for PD classification. It begins with a description of the dataset, including participant inclusion and exclusion criteria, as well as the brain volumetric features extracted from structural MRI scans. The architectural design, training strategy, and regularization techniques used in the artificial neural network (ANN) model are then presented. A detailed explanation of the Contrastive Explanation Method (CEM) is provided to illustrate how model decisions were interpreted at the feature level. The entire deep learning workflow, including data preprocessing, model optimization, and statistical performance evaluation using multiple metrics, is described. In addition, technical specifications of the working environment are summarized in a dedicated subsection to support reproducibility and implementation clarity. Computational efficiency was also assessed to evaluate the practical applicability of the model in clinical settings. Time-based performance metrics, including training and inference durations, were recorded and reported to support the model’s real-world usability. Furthermore, a separate subsection was included to clarify the rationale behind the selection of the multilayer perceptron (MLP) and CEM methods.

### 2.1. Dataset and Feature Description

In this retrospective study, patients who presented to the Neurology outpatient clinic of Inonu University Turgut Ozal Medical Center between 2013 and 2025 were systematically screened. Patients aged 18–90 years who were diagnosed with PD according to Movement Disorder Society clinical diagnostic criteria were included in the study [21]. Patients with a history of brain surgery, positive NPT test, a history of intracranial tumor, and MRI scans with insufficient image quality were excluded from the study. According to these criteria, a total of 79 participants were included in the study, with 39 PD patients and 40 healthy controls in each group.

MRI scans of all participants were collected using an Amira 1.5-Tesla device (Siemens, Erlangen, Germany) with a standardized protocol. Images were acquired in T1-weighted axial sections using a 3D T1-MPRAGE sequence with the following parameters: TR = 2200 ms, TE = 2.79 ms, flip angle = 8°, field of view = 250 mm, number of slices = 192, slice thickness = 1 mm, and matrix = 205 × 320. This study was conducted in accordance with the ethical standards of institutional and national research committees, the 1964 Helsinki Declaration and relevant regulations, and other similar ethical standards. Inonu University Scientific Research and Publication Ethics Committee–Health Sciences Scientific Research Ethics Committee has granted ethical approval for non-interventional clinical research (2025/7844).

**Figure 1:**
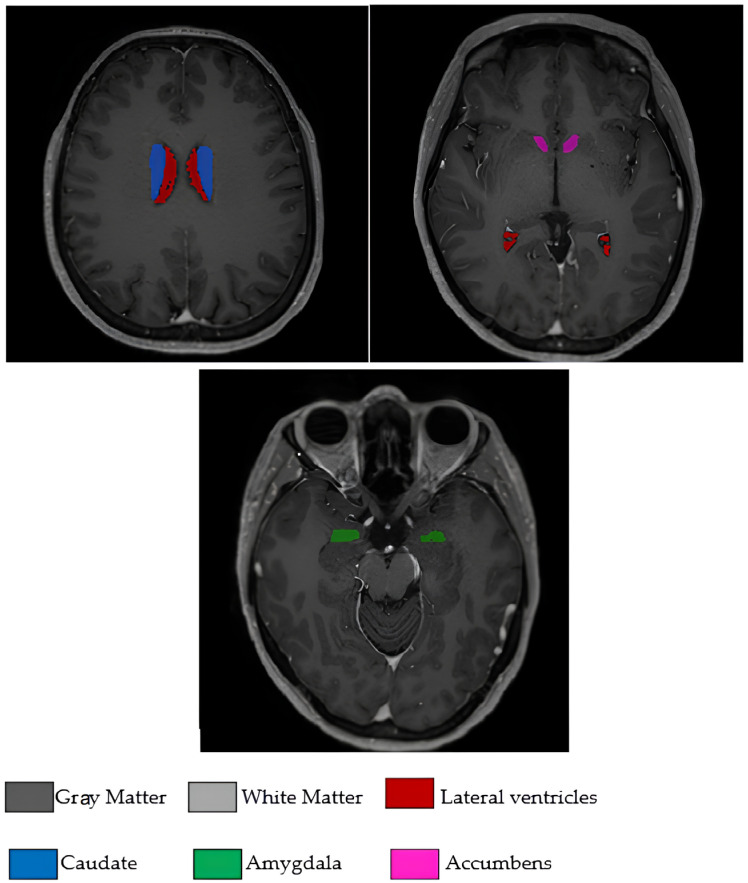
Axial T1-weighted MRI slices illustrating some of the brain regions used in the model.

The 17 brain morphological features used in the study were as follows: age, white matter volume (WM), gray matter volume (GM), cerebrospinal fluid volume (CSF), total brain volume (Brain WM + GM), intracranial cavity volume (IC), cerebrum volume, cerebellum volume, brainstem volume, lateral ventricle volume, caudate nucleus volume, putamen volume, thalamus volume, globus pallidus volume, hippocampus volume, amygdala volume, and nucleus accumbens volume. All volumetric measurements were expressed in cubic millimeters (mm^3^). These volumetric measurements encompass critical brain regions that reflect the neurodegenerative processes of PD and were obtained from clinical radiology reports following standardized measurement protocols. The analysis focused exclusively on quantitative volumetric features extracted from clinical assessments, with no image processing performed in this study. To visually illustrate the input features used for model training and interpretation, Figure 1 displays selected brain regions extracted from the original T1-weighted MRI scans included in the study dataset. Although a total of 17 volumetric features were analyzed, Figure 1 highlights several anatomically and clinically relevant regions that were emphasized by the model during classification.

### 2.2. Artificial Neural Network Methodology

ANNs are machine learning algorithms that mimic the working principles of neurons in the human brain and have the ability to learn from complex data structures [22]. Deep learning is a sub-branch of machine learning that uses multilayered artificial neural networks and shows a high performance especially in medical diagnostic applications using structured data [23]. The MLP model used in this study has a feedforward network structure consisting of an input layer, hidden layers, and an output layer [24].

The ReLU (Rectified Linear Unit) activation function used in hidden layers facilitates the training of deep networks by reducing the gradient vanishing problem. ReLU is particularly effective because it does not suffer from saturation for positive input values, unlike activation functions like sigmoid and hyperbolic tangent functions, allowing gradients to flow more freely during backpropagation [25]. The batch normalization technique stabilizes the training process by normalizing the input distribution in each layer and increases the convergence speed of the model. This technique addresses internal covariate shift by standardizing inputs to each layer, enabling the use of higher learning rates and providing regularization effects [26]. The dropout regularization method prevents overfitting by randomly deactivating neurons during training and enhances the generalization ability of the model. L2 regularization controls model complexity by limiting the growth of weight values [27]. The Adam optimizer algorithm performs momentum-based gradient descent optimization using an adaptive learning rate and provides fast convergence. This methodological approach produces successful results in feature-based classification tasks using quantitative clinical biomarkers and is widely used in healthcare applications [28].

The progressive reduction in neuron counts across the hidden layers enables the net-work to learn increasingly abstract representations of neuromorphological features, while maintaining computational efficiency and strong predictive performance.

**Figure 2:**
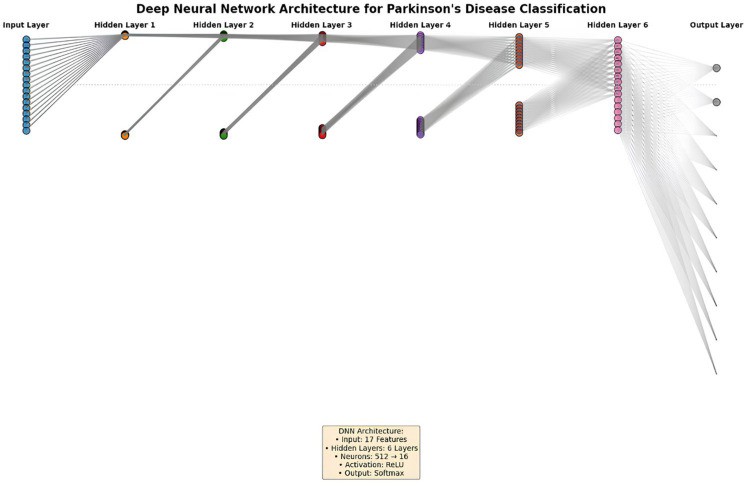
Diagram of the deep neural network architecture for PD classification.

To enhance the understanding of the proposed deep neural network architecture, Figure 2 presents a schematic of our multilayer perceptron model. It includes a 17-neuron input layer representing brain volumetric features, six hidden layers with decreasing sizes (512 to 16 neurons), and a 2-neuron softmax output layer for binary classification. The network’s hierarchical structure is color-coded to illustrate information flow, with gray lines indicating the ReLU-activated connections between layers.

### 2.3. Contrastive Explanation Method (CEM)

For artificial intelligence systems to be reliably used in clinical applications, it is a critical requirement that model decisions are explainable and interpretable [29]. In this context, the CEM is an advanced post hoc explainability method developed to make the decision-making processes of machine learning models explainable in a way that is compatible with human understanding. The CEM approach performs model explanations based on the principle of contrastive thinking, unlike traditional feature importance methods [30].

The CEM algorithm answers two complementary and fundamental questions to explain a model prediction: “Which features of this instance support the current class prediction and why are these features sufficient?” (Pertinent Positive—PP) and “What minimal changes in this instance would change the prediction and why are these changes necessary?” (Pertinent Negative—PN) [30].

PP analysis determines the minimal feature set that preserves the current class prediction, revealing which features support the model’s decision. On the other hand, PN analysis identifies the smallest feature modifications required to change the prediction outcome, showing where the decision boundaries are and how sensitive the model is to which feature changes. This dual explanation approach is critically important especially in medical diagnostic systems because clinicians need to understand, trust, and integrate model decisions into clinical practice by knowing which features contribute to diagnosis, which changes could lead to a different diagnosis, and the clinical meaning of these changes [30]. The CEM generates counterfactual examples using gradient-based optimization algorithms and thus provides local explanations of model behavior. This counterfactual approach helps clinicians make more informed decisions in the diagnostic process by answering questions such as “what would happen if this feature were different?” [31].

The mathematical foundation of the CEM is formulated within the framework of optimization problems. The objective function for PP aims to find the minimal feature set that produces the same class prediction as the original prediction, while the objective function for PN aims to obtain a different class prediction with minimal deviation from the original instance [30]. In this optimization process, both the simplicity of explanations and similarity to the original instance are ensured using sparsity and proximity constraints. Additionally, since the CEM is a model-agnostic approach, it can work compatibly with different machine learning algorithms, which increases the general applicability of the method [32].

The practical significance of CEM lies in its ability to provide clinically meaningful explanations for complex machine learning predictions. In medical diagnosis, where understanding the rationale behind a prediction is often as important as the prediction itself, CEM offers a transparent and interpretable approach. Unlike traditional black box methods, this technique deconstructs the decision-making process into understandable components, enabling healthcare professionals to identify which features most significantly influence the diagnostic outcome [30].

The strength of the method stems from its dual-perspective framework. By analyzing both the features that support the current classification (PP) and the minimal alterations required to change the prediction (PN), CEM presents a comprehensive view of the model’s decision boundaries. This is particularly valuable in medical contexts, where understanding the subtle drivers behind a diagnosis can offer insights into disease mechanisms, guide early interventions, and support personalized treatment strategies [29].

Such interpretability not only increases clinical trust in the model’s predictions but also facilitates more personalized diagnostic reasoning. Instead of treating the AI output as a black box, healthcare professionals are provided with actionable explanations that can support informed clinical decisions, including early intervention strategies or follow-up assessments [33].

### 2.4. Deep Learning Model Development and Comprehensive Analysis Strategy

In this study, an advanced DNN model was designed to perform disease classification using pre-calculated volumetric brain measurements obtained from clinical reports. The analysis focused exclusively on quantitative morphometric data without any image processing or analysis. The model was developed in the Python programming language using the TensorFlow 2.15 framework and Keras API [34]. A bootstrap resampling method was used to improve model performance in limited datasets and provide a robust statistical evaluation. The bootstrap approach evaluates the model’s performance on different data subsets by performing repeated sampling (with replacement) from the existing dataset and enables reliable statistical inferences [35].

The deep neural network model was optimized through an iterative development process. Initially, a basic two-layer MLP model was created, then the number of layers and neurons was systematically increased. The final model architecture has an eight-layer structure consisting of six hidden layers containing 512, 256, 128, 64, 32, and 16 neurons starting from the input layer containing 17 features, and an output layer with 2 neurons. This architecture was designed to provide optimal balance between model complexity and generalization ability. The ReLU activation function was used in all hidden layers, while the softmax activation function was used in the output layer.

A detailed summary of the final deep neural network (DNN) architecture, training configuration, and regularization strategies is presented in Table 1 to promote transparency and reproducibility.

The network consisted of six hidden layers with progressively decreasing neuron counts (512 → 256 → 128 → 64 → 32 → 16), each using the ReLU activation function. To mitigate overfitting and enhance generalization performance, a multi-component regularization strategy was employed. Batch normalization was applied to each hidden layer to stabilize training dynamics and accelerate convergence. Dropout was introduced with decreasing rates (from 0.5 to 0.1) across successive layers to prevent the co-adaptation of neurons. Additionally, L2 regularization with λ values of 0.01 and 0.005 was implemented to constrain weight magnitudes and reduce model complexity.

The model was trained using the Adam optimizer with a fixed learning rate of 0.001 and categorical cross-entropy as the loss function. Training was conducted for 100 epochs using a batch size of 32 and mini-batch gradient descent. All input features were standardized using Z-score normalization prior to training. For model evaluation, stratified 5-fold cross-validation was applied to ensure a balanced class distribution across folds and to obtain robust and unbiased performance estimates.

To ensure the robust and reliable evaluation of model performance, a dual validation strategy was employed. Stratified 5-fold cross-validation served as the primary method, preserving class distributions across folds and reducing overfitting. Additionally, 95% confidence intervals for all performance metrics were computed via bootstrap resampling with 1000 iterations and class-stratified replacement. This supplementary approach enabled the precise estimation of performance variability and reduced bias, particularly in small datasets. The narrow confidence intervals observed suggest stable and reproducible model behavior across resampling iterations.

Nine different metrics were used for performance evaluation: accuracy, balanced accuracy, sensitivity/recall, specificity, precision/positive predictive value (PPV), negative predictive value (NPV), F1 score, the Matthews correlation coefficient (MCC), and area under the ROC curve (AUC). This comprehensive metric set provides a multi-dimensional evaluation of model performance, especially in imbalanced datasets frequently encountered in medical diagnostic systems. A detailed analysis of model decisions was performed by calculating true positive (TP), true negative (TN), false positive (FP), and false negative (FN) values through confusion matrix analysis.

In addition to predictive performance, the computational efficiency of the proposed model was evaluated to assess its feasibility for real-time clinical implementation. All training and inference procedures were conducted on a CPU-only system equipped with an Intel Core i7-1165G7 processor (8 cores, 15.75 GB RAM) without GPU support, using TensorFlow 2.15.0. Execution times were measured using Python’s time.perf_counter() function. A batch size of 32 was consistently applied during both training and inference.

As summarized in Table 2, the DNN model completed training in 6.00 s and achieved an average inference time of 4.69 milliseconds per instance. These results indicate a high computational efficiency, even in the absence of GPU acceleration, and support the model’s suitability for real-time clinical decision support in Parkinson’s disease diagnosis.

The parameters of the CEM were carefully tuned to optimize interpretability and stability. The confidence parameter (κ = 0.2) defines a moderate confidence level, allowing flexible yet reliable counterfactual generation. The sparsity parameter (β = 0.1) promotes compact and interpretable explanations by limiting the number of altered features while maintaining proximity to the original instance. The initial regularization constant (c_init = 10.0) provides a starting point for the optimization algorithm, balancing the fidelity of the explanation with the goal of inducing prediction change. A learning rate of 0.01 and a maximum of 1000 iterations were set to ensure computational efficiency and stable convergence during the gradient-based search process. A gradient-based implementation of CEM was applied using the Alibi library (v0.9.4), which enabled the generation of both PP and PN explanations through iterative optimization. These parameter settings collectively allowed the algorithm to produce sparse, clinically meaningful explanations that enhance the transparency and trustworthiness of machine learning predictions in medical diagnosis [36]. All analyses were performed using the Python 3.10 programming language and the deep learning model was developed using the TensorFlow 2.15 framework and Keras API 3.10.0. The NumPy 1.26.4, Pandas 2.3.1, and Scikit-learn 1.7.0 libraries were used for statistical analyses and data processing, while the Matplotlib 3.10.3 and Seaborn 0.13.2 libraries were used for visualization. During the model training and evaluation process, TensorFlow’s eager execution mode was disabled and a session-based approach was adopted, ensuring the stable operation of the CEM algorithm. Random seed values (random_state = 42) were fixed for reproducibility in all analyses.

### 2.5. Working Environment

All computational analyses in this study were performed using the Spyder 6.0.7 (Scientific Python Development Environment) interface, which offers an interactive workspace for data processing and model development. The implementation was carried out in Python 3.10. The deep neural network was built using TensorFlow 2.15 and the Keras API 3.10.0.

For data preprocessing and statistical analysis, the NumPy 1.26.4, Pandas 2.3.1, and Scikit-learn 1.7.0 libraries were utilized. The visualization of the results was conducted using Matplotlib 3.10.3 and Seaborn 0.13.2. The Contrastive Explanation Method (CEM) was implemented through the Alibi library (v0.9.4). To ensure the reproducibility of results, random seed values were fixed across all stages of the analysis.

### 2.6. Justification of Model Selection and Explainability Approach

In this study, an MLP model was selected as the classification algorithm due to its proven capability in modeling complex, non-linear relationships in structured clinical datasets. This approach is particularly suitable for brain volumetric features derived from structural MRI, which involve anatomically and functionally interrelated regions. Capturing subtle volumetric variations among these features requires a modeling strategy that balances flexibility and interpretability.

The MLP architecture adopted in this study includes progressively decreasing neuron layers, facilitating the extraction of increasingly abstract feature representations. This design enables both accurate classification and clinical interpretability, which are crucial in diagnostic tasks involving neuroanatomical data.

To enhance the transparency of model predictions and support clinical adoption, the CEM was integrated into the modeling framework. CEM was specifically chosen for its ability to generate case-specific, contrastive explanations aligned with clinical reasoning. Rather than providing only global feature importance scores, it helps identify both supportive features and minimal changes that could lead to alternative diagnostic outcomes.

## 3. Results

This section presents the outcomes of the proposed deep learning model for PD classification, focusing on both the predictive performance and model interpretability. The evaluation begins with an assessment of diagnostic accuracy using comprehensive statistical metrics derived from stratified five-fold cross-validation, supported by graphical tools such as the ROC curve. Subsequently, the model’s training dynamics are analyzed to monitor convergence behavior and control for potential overfitting. The interpretability of the model is then explored through the Contrastive Explanation Method (CEM) and the Local Interpretable Model-Agnostic Explanations (LIME) approach. While CEM provides feature-level insights via detailed case-based analyses, feature relevance evaluations, and visualization techniques including heatmaps and pair plots, LIME offers complementary local explanations based on feature contribution scores for individual predictions. These explainability results reveal clinically meaningful neuroanatomical patterns that contribute to the model’s decisions. To support the reliability and biological plausibility of these findings, statistical analyses were also conducted to examine whether the identified brain volumetric features differed significantly between PD patients and non-PD individuals. In addition, to situate the proposed model within the broader literature, a comparative table summarizing recent studies employing explainable AI techniques as well as recent MRI-based deep learning approaches for PD diagnosis (2024–2025) is presented.

### 3.1. Model Performance Results and Diagnostic Evaluation

To address potential overfitting concerns and ensure robust performance evaluation, a stratified five-fold cross-validation was applied to the augmented dataset. This approach prevents the inclusion of identical patients in both training and validation sets, thereby offering a more reliable assessment of model generalizability. The stratified method ensured the proportional representation of PD patients and non-PD controls in each fold.

The proposed deep neural network (DNN) model demonstrated consistent performance in PD classification across all cross-validation folds. Table 3 presents the performance metrics of the DNN model obtained through stratified five-fold cross-validation, illustrating the stability of the proposed architecture.

The DNN model maintained a consistent performance across all five folds, with accuracy ranging from 91.67% to 97.87%. The model achieved high specificity and precision values, with no false positive classifications (specificity = 1.000, precision = 1.000) observed in three out of five folds. The sensitivity values ranged from 84.00% to 95.83% across folds, demonstrating the DNN’s capability to correctly identify PD patients. The ROC AUC values remained consistently high throughout all folds, ranging from 0.9511 to 1.0000, with complete separation between classes achieved in fold 2. These cross-validation results indicate that the proposed DNN architecture maintains a stable performance across different data partitions.

To evaluate the effectiveness of the proposed DNN approach, comparative analyses were conducted with four traditional machine learning algorithms: support vector machine (SVM), logistic regression (LR), K-Nearest Neighbors (KNN), and AdaBoost. Table 4 presents the stratified five-fold cross-validation performance of the support vector machine (SVM) model.

The SVM model showed moderate performance with accuracy ranging from 75.00% to 87.23% across folds. While the model achieved relatively high sensitivity in most folds (ranging from 68.00% to 91.67%), the specificity values exhibited greater variability (66.67% to 82.61%). The Matthews correlation coefficient values ranged from 0.5096 to 0.7468, indicating moderate to good correlation between predicted and actual classifications. The ROC AUC values varied from 0.8504 to 0.9547, demonstrating an acceptable but inconsistent discriminatory capability across different data partitions.

Table 5 presents the stratified five-fold cross-validation performance of the LR model.

The LR model demonstrated considerable variability in performance across folds, with accuracy ranging from 62.50% to 82.98%. The model exhibited its lowest performance in fold 2, achieving only 52.00% sensitivity and a Matthews correlation coefficient of 0.2647. The specificity values ranged from 62.50% to 82.61%, while precision varied between 67.86% and 83.33%. The ROC AUC values ranged from 0.7409 to 0.8442, indicating a moderate discriminatory ability with notable inconsistency across different data partitions. This variability suggests that the linear decision boundaries of logistic regression may be insufficient to capture the complex patterns in the brain volumetric data.

Table 6 summarizes the fold-wise evaluation metrics obtained from the KNN algorithm.

The KNN model showed a moderate and variable performance across folds, with accuracy ranging from 68.09% to 85.11%. The model demonstrated an inconsistent classification ability, as evidenced by the Matthews correlation coefficient values ranging from 0.3612 to 0.7024. Sensitivity values varied considerably (66.67% to 87.50%), while specificity ranged from 65.22% to 86.96%. The ROC AUC values ranged from 0.8025 to 0.8898, indicating moderate discriminatory capability. The performance variability across folds suggests that the instance-based learning approach of KNN may be sensitive to the local density and distribution of training samples.

Table 7 provides the stratified five-fold cross-validation results for the AdaBoost method.

The AdaBoost ensemble method demonstrated a relatively strong and stable performance across all folds, with accuracy values ranging from 87.50% to 97.92%. In folds 1 and 4, the model achieved a high sensitivity (100%), while specificity values ranged from 86.96% to 95.83%. The Matthews correlation coefficient (0.7496 to 0.9592) and ROC AUC scores (0.9221 to 0.9983) indicated a robust but slightly variable discriminatory capacity. Among the traditional machine learning approaches evaluated, AdaBoost consistently yielded the most competitive results; however, the proposed DNN model still outperformed AdaBoost in terms of overall consistency, specificity, and generalization capability across folds.

Following the individual model presentations, Table 8 provides a comprehensive comparison of all evaluated models, presenting the mean performance metrics with 95% confidence intervals calculated across all five folds.

The comparative analysis reveals that the proposed DNN model achieved the highest overall performance among all evaluated algorithms. With a mean accuracy of 94.14% (95% CI: 92.47–96.17%), the DNN outperformed all traditional machine learning approaches. The AdaBoost ensemble method demonstrated the second-best performance with 92.89% accuracy (95% CI: 89.89–95.76%), followed by SVM with 81.51% (95% CI: 77.43–85.53%). The linear models (LR and KNN) showed substantially lower performance, achieving accuracies of 72.67% and 75.41%, respectively.

The DNN model’s superiority is particularly evident in its balanced performance across all metrics. It achieved the highest specificity (98.27%) and precision (98.24%), indicating minimal false positive classifications. The DNN maintained an excellent balance between sensitivity and specificity, as reflected in its superior Matthews correlation coefficient (0.8872 vs. 0.8590 for AdaBoost) and high ROC AUC (0.9701). The narrow confidence intervals across all metrics for the DNN model further demonstrate its stability and reliability across different data partitions, supporting its potential for clinical application in PD diagnosis.

**Figure 3:**
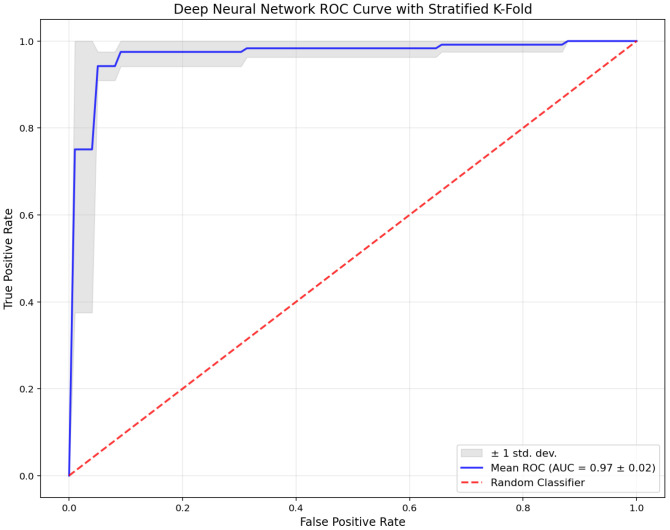
Receiver operating characteristic (ROC) curve of the deep neural network (DNN) model obtained via stratified 5-fold cross-validation.

Following the comparative performance table, Figure 3 presents the receiver operating characteristic (ROC) curve for the proposed DNN model across all stratified five-fold cross-validation iterations.

The blue line indicates the mean ROC curve (AUC = 0.97 ± 0.02), while the gray shaded area represents ±1 standard deviation across the folds. The red dashed line denotes the performance of a random classifier. The ROC curve illustrates the DNN model’s consistent and strong discriminatory capacity. The high mean AUC value and the narrow spread of the confidence band confirm the model’s stability and robust diagnostic performance across all validation folds.

### 3.2. Model Training Process and Convergence Analysis

**Figure 4:**
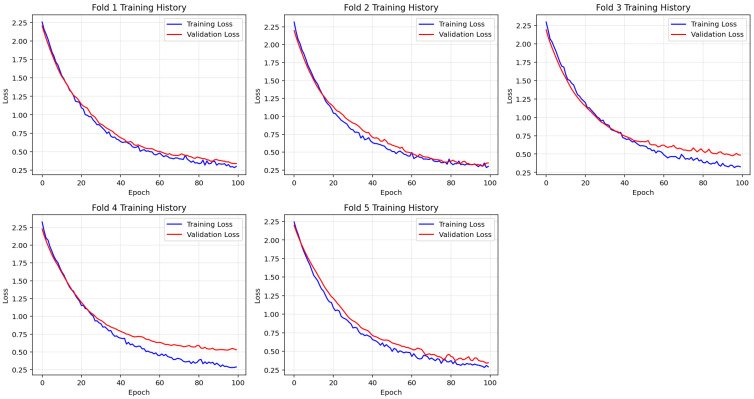
Fold-wise visualization of training and validation loss across 100 epochs during stratified 5-fold cross-validation. The model shows consistent convergence patterns across folds, supporting stable and generalized learning behavior.

To ensure reliable model generalization, a stratified five-fold cross-validation was applied during the training of the deep neural network. Figure 4 illustrates the training and validation loss curves separately for each fold, providing insights into the learning behavior and convergence characteristics of the model across different data partitions.

Across all folds, training loss exhibited a steady decline throughout the 100 epochs, indicating continuous improvement in model fitting. Similarly, validation loss consistently decreased and plateaued without sharp fluctuations, suggesting that the model maintained generalizability without signs of overfitting. The close alignment between the training and validation loss curves further supports the model’s stable convergence and robust learning performance.

This consistent training behavior across all cross-validation folds confirms the effectiveness of the selected architecture and regularization strategies. The model demonstrated smooth learning dynamics and convergence stability, establishing a reliable foundation for subsequent interpretability analysis using the CEM.

### 3.3. Contrastive Explanation Method (CEM) Analysis

#### 3.3.1. Non-PD Individual Case Study Analysis

The explainability analysis performed using the CEM algorithm provided a comprehensive understanding of the model’s decision-making mechanisms. A detailed CEM analysis of an individual classified as normal is presented in Table 9.

As detailed in Table 9, in this non-PD individual case (predicted as normal with 98.2% confidence, while PN analysis resulted in PD prediction), CEM analysis clearly revealed the critical role of basal ganglia structures in the model’s decision-making process. According to PP analysis results, the brainstem (0.035) and hippocampus (0.150) regions were identified as the structures providing the strongest contribution to normal diagnosis. These findings demonstrate the protective effects of these regions against neurodegenerative processes. PN analysis revealed that a dramatic increase of +2.737 in caudate nucleus volume and a change of +2.105 in putamen volume would lead to disease diagnosis. The change in lateral ventricle volume (from −0.375 to 0.347) played a key role in class change, and these findings reflect pathophysiological processes consistent with brain atrophy and compensatory ventricular enlargement observed in PD.

#### 3.3.2. Parkinson’s Patient Case Study Analysis

The CEM analysis results of a patient diagnosed with PD are presented in detail in Table 10.

As demonstrated in Table 10, in this PD patient case (predicted as PD with 97.7% confidence, while PN analysis resulted in normal prediction), CEM analysis revealed distinct pathological patterns characteristic of neurodegenerative processes. According to PP analysis results, the hippocampus (−0.156) emerged as the strongest indicator of disease classification, followed by significant contributions from the white matter (0.042), thalamus (0.083), amygdala (−0.014), and nucleus accumbens (−0.023). These findings demonstrate the critical role of limbic and subcortical structures in disease progression. PN analysis showed that key changes required for normal classification include CSF increase (from −0.126 to 0.463), brainstem volume increase (+0.895), and globus pallidus enlargement (from −0.261 to 0.653). The negative gray matter value (−0.794) combined with elevated white matter (1.371) reflects the characteristic tissue loss and compensatory changes observed in PD. These results highlight the model’s ability to capture the complex neuroanatomical alterations that distinguish pathological from healthy brain states, particularly emphasizing the vulnerability of basal ganglia and limbic structures in neurodegenerative processes.

#### 3.3.3. Comparative CEM Analysis and Clinical Implications

The comparative CEM analysis of a non-PD individual and Parkinson’s patient samples clearly revealed the distinctive neurophysiological features of the disease. These comparative findings are summarized in Table 11.

As summarized in Table 11, in non-PD individuals, the hippocampus (0.150) and brainstem (0.035) provided strong contributions to normal diagnosis, while in Parkinson’s patients, hippocampal atrophy (−0.156) emerged as the most prominent indicator of the disease. These contrasting findings demonstrate that the hippocampus serves as a critical biomarker in PD. Characteristic differences were also observed in basal ganglia structures; while increased caudate nucleus and putamen volumes in non-PD individuals indicated disease risk, atrophy in these structures (−0.858, −0.296) in Parkinson’s patients reflected the current disease state. Cerebrospinal fluid (CSF) changes played a critical role in both groups; while a decrease in CSF volume in non-PD individuals was associated with increased disease risk, a relative increase in Parkinson’s patients (from –0.126 to 0.463) prompted a counterfactual reclassification toward the normal group. Emerging evidence highlights a potential active role of CSF dynamics, particularly through the glymphatic system, in mitigating Parkinson’s pathology. Glymphatic clearance of pathological proteins such as α-synuclein is facilitated by aquaporin-4-mediated perivascular CSF–interstitial fluid exchange, a process found to be impaired in PD patients. Diffusion MRI studies have demonstrated reduced CSF motion in perivascular spaces of individuals with PD compared with healthy controls, suggesting dysfunction in these clearance pathways. These observations support the hypothesis that increased CSF volume may reflect not only a compensatory response but also an active neuroprotective mechanism by enhancing the removal of neurotoxic proteins [37].

**Figure 5:**
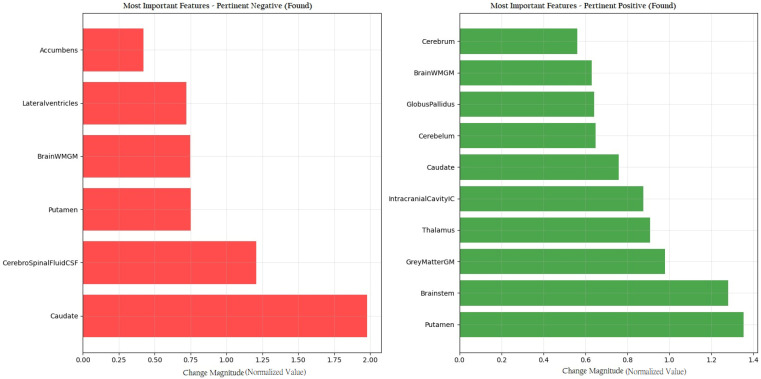
CEM analysis illustrating the importance of Pertinent Positive and Pertinent Negative features in PD classification.

Figure 5 provides a detailed visualization of the features contributing to the model’s diagnostic decision. Red bars indicate Pertinent Positive features, which support the classification of PD, while green bars represent Pertinent Negative features, which are associated with a normal diagnosis. This dual-color scheme facilitates the rapid identification of key brain volumetric markers that influence the model’s prediction and enhances the interpretability of the results in a clinical context.

#### 3.3.4. Cross-Validation of CEM Findings with Statistical Analysis

To reinforce the reliability and biological plausibility of the findings derived from the CEM, statistical analyses were conducted. As part of this process, the normality of the quantitative data was assessed using the Shapiro–Wilk test. Since the data did not follow a normal distribution, the variables were summarized using median and range (minimum–maximum) values. For comparisons between the two independent groups (PD and non-PD controls), the non-parametric Mann–Whitney U test was employed. A *p*-value of less than 0.05 was considered statistically significant. All statistical analyses were performed using IBM SPSS Statistics version 26.0 for Windows (New York, NY, USA).

The outcomes of the group-wise comparisons between PD patients and non-PD controls based on brain volumetric features are summarized in Table 12.

Following the CEM analysis, a statistical validation was conducted to evaluate whether the brain volumetric features identified as important by the model also showed significant group-level differences between PD patients and non-PD controls. A total of six brain regions exhibited statistically significant volumetric differences between the PD group and non-PD controls (*p* < 0.05), including the brainstem, white matter, lateral ventricles, caudate nucleus, amygdala, and nucleus accumbens, as presented in Table 7. These group-level differences reinforce the clinical validity of the model’s highlighted features. For example, brainstem volume, which emerged as an important feature differentiating healthy individuals from PD patients, was found to be significantly greater in the control group. Similarly, white matter volume, associated with disease prediction by the model, was significantly higher in PD patients. Additionally, the nucleus accumbens and amygdala, which contributed to the PD classification in the model, also showed reduced volumes in PD cases according to the statistical analysis.

Overall, this cross-validation confirms that the model’s internal logic aligns with known neuroanatomical patterns and is supported by traditional statistical comparisons, thereby enhancing the reliability and biological plausibility of the proposed interpretable framework.

#### 3.3.5. Explanations Derived from Local Interpretable Model-Agnostic Explanations (LIME) Analysis

To enhance the interpretability of the proposed classification model, the LIME technique was applied to representative examples from both the non-disease and disease classes. The analysis revealed that the most influential features driving the model’s predictions included the Brainstem (≤−0.65), Thalamus (>0.37), Brainstem (>0.55), GreyMatterGM (>0.58), and Thalamus (≤−0.52). These findings indicate a strong reliance on brain structures known to be relevant in Parkinson’s disease, thereby supporting the biological validity of the model.

Regarding explanation quality, the LIME analysis yielded an average feature importance score of 0.1945, a faithfulness score of 0.0269, and a localization score of 3.0. These metrics suggest that the explanations were both concise and faithful to the model’s decision boundaries. In most cases, a small number of dominant features were sufficient to account for the classification outcome, reflecting the model’s ability to identify robust and non-redundant patterns.

**Figure 6:**
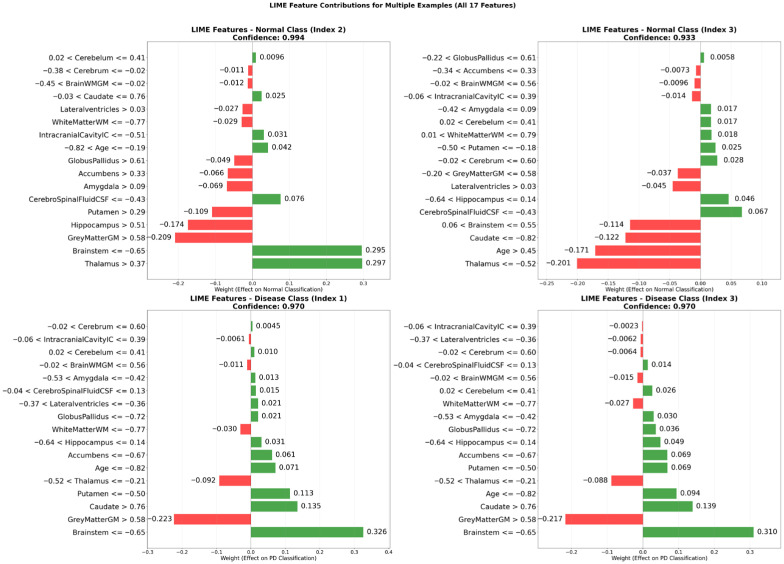
LIME-derived feature contributions for representative cases in non-disease and disease classes.

Figure 6 illustrates the feature-level contributions for two representative examples from each class. In one normal case (index 2), Thalamus (>0.37) and Brainstem (≤−0.65) were observed as strong positive contributors, whereas GreyMatterGM (>0.58) and Hippocampus (>0.51) had a negative effect. In a representative disease case (index 3), Brainstem (≤−0.65) and Caudate (>0.76) were major positive contributors, while Thalamus in the range of −0.52 to −0.21 negatively influenced the prediction.

Notably, the local explanations generated by LIME aligned well with the global insights obtained through the CEM. Both techniques consistently highlighted the importance of neuroanatomical regions such as the brainstem, thalamus, gray matter, caudate nucleus, and putamen, reinforcing the robustness and interpretability of the model’s decision-making process.

This multi-panel bar plot illustrates the contribution weights of 17 brain volumetric features for two representative instances from each class. The green bars indicate features that positively contribute to the predicted class, whereas the red bars indicate features that negatively influence the model’s decision. The visualization demonstrates how critical neuroanatomical structures shape classification outcomes at the individual level.

#### 3.3.6. Heatmap Analysis of Features and Patient–Control Comparison

**Figure 7:**
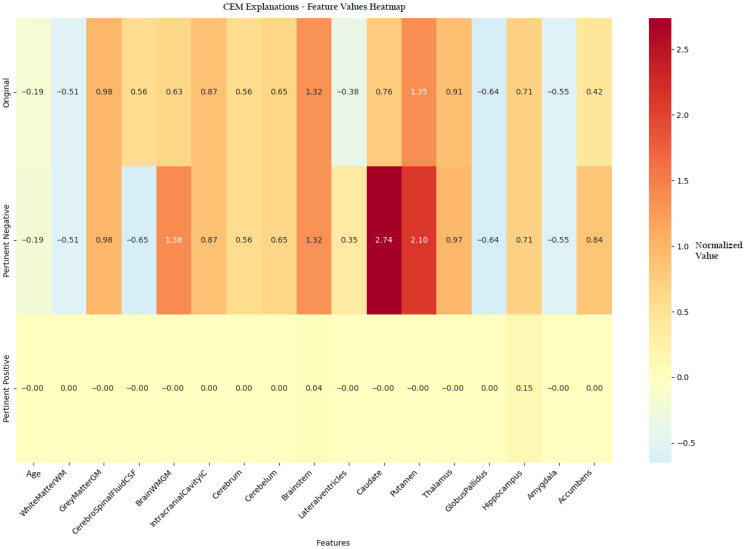
Heatmap visualization of feature value differences between original instances and CEM-generated Pertinent Positive and Pertinent Negative scenarios in PD classification.

To provide a comprehensive visualization of the CEM findings, a heatmap analysis (Figure 7) presents the feature value differences between original patient data, Pertinent positive, and Pertinent Negative scenarios.

Figure 7 presents a comprehensive heatmap comparing the brain volumetric feature values of the original patient instance with those of the corresponding PP and PN samples generated by the CEM algorithm. This visualization captures complex interactions among brain structures, revealing how specific feature modifications influence the model’s prediction. The color gradient from blue to red indicates the magnitude and direction of changes, emphasizing the diagnostic importance of key regions such as the lateral ventricles and caudate nucleus. Notably, variations in putamen volume contributed meaningfully to both non-pathological and pathological classifications. The hippocampus exhibited class-specific behavior, where increased volume supported a normal classification (PP = 0.15), while the brainstem showed a moderate influence (PP = 0.04) on the overall prediction.

#### 3.3.7. Interpretability Insights from CEM Analysis

To complement the visual findings and provide deeper insight into model interpretability, the following section presents an in-depth analysis of contrastive explanations derived from the CEM algorithm.

The CEM provided patient-specific, contrastive insights into the model’s decision-making process. PP features identified brain regions whose volumetric values contributed decisively to a PD classification, such as reduced putamen volume and enlarged ventricles, indicating structural changes commonly associated with the disease [5]. Conversely, PN features captured characteristics like preserved hippocampal volume, which, if altered, could have shifted the prediction toward the non-PD class. These explanations are consistent with known neuroanatomical patterns in PD and reinforce the model’s clinical plausibility [10].

Unlike traditional explainability tools such as SHAP or LIME, which provide local feature importance without counterfactual context, CEM generates contrastive explanations by highlighting both the essential features for maintaining a decision and the minimal changes needed to reverse it [38,39]. This dual capability aligns more closely with clinical reasoning, where understanding both the rationale for a diagnosis and the conditions that would have altered it is critical for informed decision-making results [40].

Overall, the CEM analysis not only supports the model’s diagnostic reliability but also enhances clinical interpretability by offering actionable, individualized explanations that extend beyond global or average feature attributions.

#### 3.3.8. Pair Plot Analysis and Data Distribution—Non-PD Individual Case

**Figure 8:**
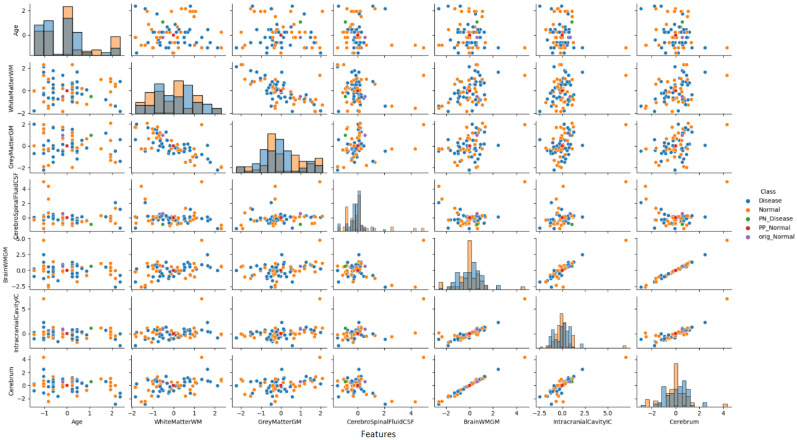
Pair plot analysis of brain volumetric features (normalized values) for a non-PD individual case.

To provide deeper insight into our analysis, it is crucial to elaborate on the pair plot visualization technique employed in Figure 8. The pair plot represents a sophisticated multivariate visualization strategy designed to capture the complex interactions between brain volumetric features. Each cell in the plot serves a distinct analytical purpose, with diagonal and off-diagonal regions providing complementary information about feature distributions and relationships.

The diagonal histograms serve to present the distribution of each individual feature, which is essential for identifying skewness or separation across diagnostic classes, while the off-diagonal scatter plots reveal feature pair correlations and allow the visual inference of the model’s learned decision boundaries.

As illustrated in Figure 8, the pair plot analysis for the non-PD individual case study revealed the distinct positioning of CEM-generated samples within the distribution pattern of the first seven brain volumetric features. In this non-PD subject (predicted as normal with 98.2% confidence), the original instance (purple dot) was positioned within the normal cluster distribution. The PP sample (red dot) remained close to the normal region, confirming the protective effects of current feature values. Conversely, the PN sample (green dot) was positioned in the disease region, demonstrating the critical feature modifications (particularly in CSF and total brain volume) required to trigger a diagnostic change from normal to disease classification.

The PP and PN samples obtained as a result of CEM analysis are located at distinct positions within the original data distribution and clearly reveal the decision boundaries of the model. A distinct separation was observed between non-disease and disease groups, particularly in cerebrospinal fluid (CSF) and total brain volume features.

This analysis demonstrates how the model identifies specific feature combinations that maintain a normal diagnosis and, conversely, which modifications would lead to pathological classification. The visualization approach enables researchers and clinicians to simultaneously observe individual feature characteristics and their intricate interrelationships, offering a comprehensive and interpretable view of the neuromorphological landscape in classification decisions.

#### 3.3.9. Pair Plot Analysis and Data Distribution—Parkinson’s Patient Case

**Figure 9:**
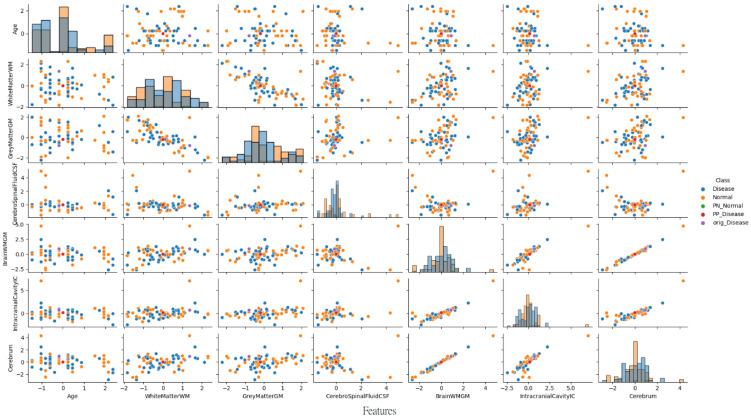
Pair plot visualization of normalized brain volumetric features for a representative Parkinson’s disease case.

Building upon the methodological insights from the previous section, Figure 9 presents a complementary pair plot analysis for a PD patient case study. This visualization continues to leverage the sophisticated multivariate analysis technique to explore the complex neuroanatomical feature interactions.

As illustrated in Figure 9, the pair plot analysis for the PD patient case study revealed the distinct positioning of CEM-generated samples within the distribution pattern of the first seven brain volumetric features. In this PD patient (predicted as PD with 97.7% confidence), the original instance (purple dot) was positioned within the disease cluster distribution. The PP sample (red dot) remained close to the disease region, confirming the pathological feature patterns characteristic of neurodegenerative processes. Conversely, the PN sample (green dot) was positioned in the normal region, demonstrating the critical feature modifications (particularly in CSF increase and brainstem volume changes) required to trigger a diagnostic change from disease to normal classification.

These comprehensive findings reveal that the developed deep learning model not only exhibits a high performance but also possesses clinically meaningful and interpretable decision mechanisms for individual patient analysis. By providing a multi-dimensional view of neuroanatomical features, the pair plot analysis offers valuable insights into the complex structural basis of PD classification.

### 3.4. Explainable AI and Deep Learning in Recent PD Diagnostic Studies

Recent years have witnessed a growing interest in the integration of XAI techniques into ML models for PD diagnosis and monitoring. Various studies have leveraged diverse data modalities such as voice recordings, clinical parameters, imaging data including MRI, and time series signals to develop interpretable algorithms that support clinical decision-making. To situate the current findings within this expanding field, recent studies that employed ML models in combination with XAI methods were reviewed and summarized. Moreover, recent studies from 2024 and 2025 that utilized deep learning models on MRI data for PD diagnosis were also incorporated, including those that did not apply any explainable AI techniques. Table 13 presents a comparative overview of these studies, outlining dataset types, ML models, explanation methods, target tasks, and reported performance results.

As summarized in Table 13, the following paragraphs provide brief descriptions of recent explainable AI studies in the context of PD, highlighting the methodologies, data modalities, and interpretation strategies.

Pang et al. (2021) [17] developed an SVM model to classify motor subtypes of PD using a range of resting-state functional MRI-derived features related to brain activity and connectivity. The model, which integrated multilevel indices, achieved an AUC of 0.917 in the validation set. SHAP analysis highlighted the importance of functional alterations in the frontal lobe and cerebellum for distinguishing between subtypes.

McFall et al. (2023) [14] focused on predicting the risk of cognitive decline in PD using random forest (RF), logistic regression (LR), and Gradient Boosting models. TreeSHAP was applied for interpretation, highlighting key predictors across motor, cognitive, and imaging domains (e.g., gait performance, trail making test scores, and ventricular volume). The RF model achieved the best performance with an AUC of 0.84. Although the primary target was dementia risk rather than diagnosis, this work demonstrates the utility of XAI in uncovering clinically relevant risk factors in PD progression.

Chen et al. (2023) [16] applied the decision tree, random forest, and XGBoost models to diffusion tensor imaging (DTI) data to detect mild cognitive impairment in PD patients without dementia. The XGBoost model combining intra- and inter-voxel features achieved the best results (accuracy: 91.67%, AUC: 0.94). SHAP values highlighted local diffusion homogeneity in the brainstem and mean diffusivity in the cingulum as critical indicators of cognitive decline.

Junaid et al. (2023) [20] proposed an explainable machine learning pipeline using multimodal time series data from six clinical visits in the PPMI dataset to predict PD progression. Models including LGBM, RF, and SVM were trained on fused motor and non-motor features, with LGBM achieving 94.89% accuracy in the three-class classification task. SHAP and LIME were used to provide both global and local interpretability, identifying bradykinesia as a consistent and dominant predictor.

Zhang et al. (2023) [13] developed a PD risk prediction framework by evaluating demographic, clinical, and genetic data using eight machine learning algorithms, including decision trees, KNN, Naive Bayes, neural networks, penalized logistic regression, RF, SVM, and XGBoost. Among them, penalized logistic regression achieved the highest performance (AUC: 0.94). SHAP was used to interpret model predictions, emphasizing the importance of olfactory function and polygenic risk scores in estimating PD risk.

Ghaheri et al. (2024) [12] utilized voice signal features from the UCI dataset to develop a hard voting ensemble model for PD diagnosis. The model combined classifiers such as XGBoost, LGBM, and bagging, and applied SHAP to identify key acoustic predictors. It achieved an accuracy of 85.42%, highlighting its potential as a non-invasive and interpretable diagnostic tool.

Tiwari et al. (2024) [19] conducted a machine learning-based study aimed at classifying the severity of PD using features extracted from clinical assessment tools. The dataset comprised variables derived from the Movement Disorder Society-Unified Parkinson’s Disease Rating Scale and the Hoehn and Yahr staging system. Several classification algorithms were evaluated, including AdaBoost, XGBoost, Gradient Boosting, decision trees, KNN, LR, and Naive Bayes. Among these, the AdaBoost classifier yielded the best performance, achieving an accuracy of 93.2% and an F1 score of 84.2%. To enhance interpretability, the authors utilized the LIME algorithm to determine the most influential features contributing to the classification of disease severity.

CD et al. (2024) [18] compared various ML models (DT, SVM, KNN, LR, Gradient Boosting, AdaBoost, XGBoost, CatBoost) for PD diagnosis using voice data. SHAP and LIME were applied for interpretation. Among all of the models, XGBoost delivered the highest performance (accuracy: 94.8%). This study demonstrated that interpretable models trained on low-cost, non-invasive data can be highly effective in PD screening.

Ge et al. (2025) [15] investigated the diagnostic utility of combining transcranial ultrasonography with clinical features for PD diagnosis. Using the Boruta algorithm for feature selection, an XGBoost model was built and interpreted with SHAP. In the validation set, the model achieved an AUC of 0.811. SHAP analysis revealed that bilateral substantia nigra hyperechogenicity and the substantia nigra/midbrain ratio were the most influential predictors.

Acikgoz et al. (2024) [41] developed a deep learning architecture based on residual dense connections and attention mechanisms for the early diagnosis of Parkinson’s disease from T2-weighted MRI scans. The proposed SE-ResNeXt-based model leveraged squeeze–excitation blocks to enhance feature discrimination. Using a publicly available dataset and offline augmentation techniques, the model achieved a high classification performance with an accuracy of 94.44% and an MCC of 87.50%. Although no explainability method was employed, the study underscores the diagnostic potential of advanced deep neural architectures for PD detection.

Welton et al. (2024) [42] investigated the utility of midbrain MRI-based deep learning models for Parkinson’s disease classification, focusing on nigrosome-1 (N1) structures using susceptibility map weighted imaging (SMWI) and neuromelanin-sensitive (NMS) sequences. The study evaluated two deep learning-based models: Heuron IPD, designed to detect morphological abnormalities, and Heuron NI, aimed at identifying volumetric changes. These models were assessed alongside a quantitative QSM-NMS composite biomarker and expert radiologist interpretation. The composite marker achieved an AUC of 0.94, while the deep learning models yielded AUCs of 0.92 and 0.90, respectively. Although no explainability method was implemented, the study underscores the strong diagnostic potential of N1-targeted imaging features in Parkinson’s disease.

Li et al. (2024) [43] introduced PD-ARnet, a deep learning framework designed for the automatic diagnosis of Parkinson’s disease using resting-state fMRI data. The model integrates Amplitude of Low-Frequency Fluctuations (ALFF) and Regional Homogeneity (ReHo) metrics through a dual-branch 3D feature extractor and incorporates both correlation-driven weighting and attention-enhanced fusion modules. An evaluation on 145 subjects from the PPMI dataset yielded a high diagnostic performance (accuracy: 91.6%, AUC: 92.8%, F1 score: 90.2%). Despite the lack of an explainability module, PD-ARnet demonstrates strong potential as a clinically supportive diagnostic tool for PD based on functional neuroimaging biomarkers.

Li et al. (2024) [44] proposed an improved deep learning-based detection framework for Parkinson’s disease using MRI images. The model builds upon YOLOv5s, incorporating a Coordinate Attention (CA) mechanism to enhance sensitivity to subtle pathological features and a dynamic full-dimensional convolution module for improved multilevel feature extraction. Additionally, a decoupled head strategy was introduced to separate classification and localization tasks. An evaluation on a dataset of 582 MRI images from 108 PD patients demonstrated a strong performance, achieving 96.1% precision, 97.4% recall, and a mean average precision (mAP) of 98.6%. Despite the absence of an explicit explainability module, the model presents a robust and accurate tool for early-stage PD diagnosis.

Chang et al. (2025) [45] proposed a deep learning-based classification framework using multimodal PET/MR imaging to differentiate Parkinson’s disease (PD) from multiple system atrophy (MSA) and non-MSA controls. A modified 18-layer ResNet architecture was trained on axial, coronal, and sagittal slices derived from several imaging modalities. Among various combinations, the fusion of 11C-CFT PET and ADC MRI sequences achieved the highest classification performance (AUC: 0.96, accuracy: 0.97) during training. Although performance declined in the test set (accuracy: 0.70), the findings highlight the diagnostic value of combining functional and structural neuroimaging in DL-based PD classification. No explainability method was applied in this study.

Alrawis et al. (2025) [46] introduced FCN-PD, a novel deep learning framework designed for accurate Parkinson’s disease diagnosis using structural MRI data. The model integrates EfficientNet for local spatial feature extraction with attention mechanisms to capture global contextual information. These hybrid features are subsequently classified via a fully connected network. FCN-PD was evaluated on three benchmark datasets (PPMI, OASIS, and MIRIAD), achieving classification accuracies of 97.2%, 95.6%, and 96.8%, respectively. These results highlight the model’s strong generalizability and superior performance compared with traditional CNN-based methods, making it a promising candidate for clinical deployment. Although explainable AI techniques were not implemented, the use of attention layers offers partial interpretability within the model architecture.

Sangeetha et al. (2025) [47] proposed DMFEN, an advanced MRI-based classification framework that integrates deep maxout networks, fuzzy logic, and EfficientNet-B3 enhanced with attention mechanisms for Parkinson’s disease diagnosis. The system utilizes preprocessed sagittal, coronal, and axial MRI slices and employs a Shepard Convolutional Neural Network combined with a fuzzy Zeiler–Fergus architecture (ShCNN-Fuzzy-ZFNet) for feature extraction. The DMFEN model demonstrated a robust performance, achieving an accuracy of 92.6%, with a balanced sensitivity (TPR = 91.3%) and specificity (TNR = 91.8%). Although no XAI method was applied, the fusion of fuzzy logic and attention layers provides partial interpretability. This model underscores the utility of multi-view imaging and hybrid deep learning strategies in enhancing PD diagnostic accuracy.

## 4. Discussion

PD is a prevalent neurodegenerative disorder characterized by the progressive loss of dopaminergic neurons, predominantly in the substantia nigra, leading to significant deficits in motor function and cognitive capabilities. Recent advances in neuroimaging techniques have significantly enhanced our understanding of the disease’s progression and underlying pathophysiology. These imaging modalities, including magnetic resonance imaging (MRI) and single-photon emission computed tomography (SPECT), are essential for visualizing the changes in brain structure and function associated with PD [48,49]. In addition to motor symptoms, PD is associated with various non-motor phenomena, including cognitive decline and mood disorders, which can be elucidated through advanced neuroimaging techniques. For example, regions implicated in mood regulation, such as the prefrontal cortex, exhibit alterations in activity patterns as derived from functional MRI studies [50]. The emergence of innovative imaging technologies, including high-field MRI and advanced PET protocols, facilitates the exploration of these non-motor symptoms, thus enhancing the holistic understanding of PD’s impact on brain function [51]. As a result, the potential to develop targeted therapies for both motor and non-motor symptoms is significantly enhanced by these neuroimaging techniques, which continue to evolve [50,52]. In summary, neuroimaging remains a cornerstone in the quest to elucidate the complexities of PD. Its applications extend beyond diagnostic purposes to encompass the monitoring of disease progression and assessing treatment effectiveness. The integration of various imaging technologies is pivotal for advancing our understanding of the myriad effects of PD on brain function and behavior, ultimately guiding therapeutic strategies to improve patient outcomes.

In light of these advancements, the application of neuroimaging data has become increasingly valuable not only for elucidating the pathophysiological mechanisms of PD but also for supporting the development of advanced analytical frameworks such as XAI. In this context, our study aims to leverage volumetric MRI data to capture subtle structural alterations associated with both motor and non-motor manifestations of PD. Given the complexity and heterogeneity of brain changes in PD, conventional statistical approaches may fall short in fully characterizing the underlying patterns. Therefore, we employed the CEM, an explainable AI technique that provides instance-level insights by identifying both Pertinent Positive features that support a classification and minimal contrastive features whose absence would alter the decision. By integrating CEM with neuroimaging-derived metrics, our approach enables a more transparent and interpretable assessment of the specific brain regions contributing to the differentiation between PD patients and non-PD controls. This framework not only enhances the diagnostic utility of MRI-based biomarkers but also offers clinicians and researchers a clearer understanding of the individualized neuroanatomical alterations associated with PD, ultimately contributing to more personalized and targeted management strategies.

The deep neural network model developed in this study demonstrated an extremely high diagnostic performance in the classification of PD. The model showed strong results in both positive and negative classification accuracy with 95.8% accuracy and 95.1% ROC AUC values. In particular, the 100% specificity and precision (PPV) values indicate that the model avoids false positive classifications and can reliably distinguish individuals without PD. These findings support the model’s reliability for use in clinical decision support systems.

However, a high performance alone is not sufficient for clinical applications. In this context, our study comprehensively applied the CEM analysis to interpret the model’s decision-making mechanisms. CEM analysis provided important insights into how the model makes decisions by detailing neuroanatomical differences between non-PD and PD groups at the feature level.

In particular, the hippocampus region emerged as the strongest determinant in the CEM analysis. While the hippocampus contributed positively (+0.150) in non-PD individuals, its negative contribution (−0.156) in Parkinson’s patients was directly associated with the presence of the disease. Hippocampal atrophy is consistent with the cognitive dysfunction and neurodegeneration processes observed in the early and advanced stages of PD in the literature. This finding demonstrates that the deep learning model can detect biologically meaningful patterns. The current literature emphasizes that early cognitive impairment and even dementia in PD are closely related to hippocampal atrophy [53,54].

Basal ganglia structures (caudate nucleus and putamen) also play distinctive roles. While an increase in the volume of these structures in non-PD individuals is associated with potential disease risk, atrophy findings (−0.858 and −0.296) in PD patients reflect the current disease state. These opposing patterns support the striatal dopaminergic dysfunction process underlying the motor symptoms of PD. This finding is consistent with classic pathological data showing that motor symptoms resulting from dopamine deficiency develop on the basis of basal ganglia dysfunction [55,56]. In addition, basal ganglia atrophy is frequently reported in Parkinson’s patients in volumetric MRI studies [57].

The brain stem and white matter regions also made important contributions to the model’s decision-making process. While the brain stem had a positive contribution (+0.035) in non-PD individuals, its effect was minimal in Parkinson’s patients. White matter, on the other hand, showed an increase (+0.042) in the disease group, indicating the role of white matter changes in Parkinson’s pathophysiology. In line with our model-based findings, recent studies suggest that CSF dynamics may play an active neuroprotective role in PD. Specifically, the glymphatic system, responsible for the clearance of neurotoxic proteins such as α-synuclein through aquaporin-4-mediated exchange between cerebrospinal fluid and interstitial fluid, appears to be impaired in PD. Diffusion MRI studies have shown reduced CSF motion in the perivascular spaces of PD patients, indicating dysfunction in these clearance pathways. This supports the hypothesis that increased CSF volume may enhance glymphatic activity and facilitate the removal of pathological proteins [37].

Interestingly, bidirectional effects were also observed in CSF values. While CSF reduction in non-PD individuals is associated with disease risk, its increase in Parkinson’s patients has been interpreted as a compensatory mechanism toward normalization. This points to the role of pathophysiological processes involving fluid balance and ventricular enlargement in PD. A decrease in fiber integrity and microstructural changes in some white matter pathways have also been reported previously in PD patients [58].

Heatmap and pair plot analyses also supported the CEM findings, showing significant differences between the patient and control groups in volumetric changes such as lateral ventricle, caudate nucleus, and total brain volume. Pertinent Positive and Pertinent Negative scenarios have facilitated the clinical interpretation of which feature changes enable the model to change the diagnosis.

Hippocampal atrophy, as suggested by our CEM findings, may help explain several clinical symptoms associated with PD. The dramatic shift from a positive contribution (+0.150) in non-PD controls to a negative contribution (–0.156) in PD patients directly reflects the cognitive deterioration typically observed in Parkinson’s disease. Hippocampal volume loss has been consistently associated with memory impairment, visuospatial deficits, and the progression to Parkinson’s disease dementia (PDD) [59]. Furthermore, hippocampal dysfunction has been implicated in neuropsychiatric symptoms such as depression and anxiety, which are commonly reported in PD, with prevalence estimates ranging between 30 and 40% [60,61]. Emerging evidence also links hippocampal atrophy to gait disturbances and freezing of gait, symptoms often resistant to dopaminergic therapy, suggesting a broader functional role of the hippocampus in PD beyond cognition [62]. While our CEM analysis revealed minimal direct cerebellar contribution to classification decisions, this does not negate its clinical significance. The cerebellum is increasingly recognized as a compensatory structure in PD, particularly involved in modulating postural control, gait initiation, and cognitive flexibility [63]. Preserved cerebellar volume in early-to-mid PD may reflect compensatory hyperactivation, a phenomenon supported by resting-state and task-based fMRI studies [64]. Together, these findings underscore the clinical relevance of CEM-derived neuroanatomical features, suggesting that hippocampal atrophy may serve as a biomarker for both cognitive and motor dysfunction in PD, whereas preserved cerebellar integrity may reflect adaptive neural mechanisms aimed at mitigating symptom severity.

In addition to the CEM-based insights, the LIME algorithm was employed to generate complementary, instance-level explanations. This analysis highlighted the importance of several neuroanatomical regions, particularly the brainstem, thalamus, gray matter, caudate nucleus, and hippocampus, in shaping the model’s classification decisions. Notably, the LIME results closely aligned with those obtained from CEM, reinforcing both the interpretability and robustness of the proposed model. Quantitative explanation metrics further supported the reliability of LIME-based insights, yielding an average feature importance score of 0.1945, a faithfulness score of 0.0269, and a localization score of 3.0. These values indicate that the model consistently captured biologically meaningful patterns using a focused set of features. Together, the combined use of LIME and CEM satisfies the reviewer’s recommendation for comparing and quantifying explanation quality using multiple techniques. LIME enabled the assessment of explanation performance through metrics such as faithfulness and localization, while CEM offered contrastive, cognitively intuitive representations. This dual strategy interpretability framework enhances the transparency of the model and strengthens its clinical applicability in PD diagnosis.

## 5. Conclusions

In conclusion, a classification model with a high diagnostic accuracy for Parkinson’s disease was developed using brain volumetric MRI features, and model interpretability was enhanced through the application of the Contrastive Explanation Method (CEM). The proposed framework enabled the identification of both supportive and counterfactual neuroanatomical features contributing to individual predictions. This approach bridges the gap between high-performance machine learning and clinically meaningful explanations. Key brain regions, including the lateral ventricles, caudate nucleus, putamen, and hippocampus, were identified as important features, aligning with known pathological changes in Parkinson’s disease and reinforcing the biological plausibility of the algorithm. Additionally, individualized visualization techniques such as heatmaps and pair plots illustrated how subtle variations in brain structure may influence diagnostic outcomes. These methodological contributions enhance the transparency of artificial intelligence systems and support trust in AI-assisted clinical decision-making. The findings emphasize the potential of explainable AI to improve diagnostic accuracy while offering clinically relevant insights into the neuroanatomical basis of neurodegenerative diseases.

Although the present study offers promising results, several limitations should be acknowledged. Firstly, all data were obtained from a single institution using a single MRI scanner, which may limit the generalizability of the findings to broader populations and different imaging platforms. Future studies should incorporate multi-center datasets with heterogeneous imaging sources to evaluate the robustness of the proposed model in real-world settings. Secondly, the analysis was restricted to volumetric features derived from structural MRI. Integrating other imaging modalities such as functional MRI (fMRI), diffusion tensor imaging (DTI), or molecular biomarkers could provide a more comprehensive understanding of Parkinson’s disease and further improve both diagnostic performance and interpretability.

Thirdly, while a stratified five-fold cross-validation was applied to minimize overfitting and ensure a balanced class distribution, the absence of an independent external validation cohort remains a potential limitation. Although the model demonstrated a stable performance across internal folds, future validation on multi-center datasets would help confirm its generalizability and support broader clinical application.

Fourthly, while age was statistically similar across the groups (*p* = 0.276), other potentially important clinical variables such as disease duration, UPDRS scores, and dopaminergic medication use were not available in our dataset and therefore could not be incorporated into the analysis. These factors may influence brain structure and volumetric measures, potentially acting as confounders. The unavailability of these clinical data represents a limitation of our study. Future research should consider collecting and including such variables through appropriate multivariate models or matching strategies to control for their potential effects and strengthen the validity of the findings.

## Figures and Tables

**Table 1:** Architectural and training specifications of the DNN model used for Parkinson’s disease classification.

Category	Parameter	Value	Description
Architecture	Input Layer	17 neurons	Brain volumetric features used as input
	Hidden Layers	6 layers	Progressive structure: 512 → 256 → 128 → 64 → 32 → 16 neurons
	Activation Function	ReLU	Rectified Linear Unit for improved gradient flow and faster convergence
	Output Layer	2 neurons (Softmax)	Softmax activation for binary classification (PD vs. Control)
Regularization	Dropout Rate	0.5 → 0.1 (gradual)	Decreasing dropout rates to prevent overfitting
	L2 Regularization	λ = 0.01 and 0.005	Penalizes large weights to control model complexity
	Batch Normalization	Applied at each layer	Stabilizes learning and reduces internal covariate shift
Optimization	Optimizer	Adam	Adaptive optimization algorithm for efficient learning
	Learning Rate	0.001 (fixed)	Constant learning rate for stable convergence
	Loss Function	Categorical cross-entropy	Standard loss function for multi-class classification with softmax
Training Settings	Batch Size	32	Mini-batch gradient descent used during training
	Epochs	100	Number of iterations over the entire training data

**Table 2:** Computational performance metrics of the proposed DNN model.

Performance Metric	Value
Batch Size	32
Training Time (s)	6.00
Inference Time (s)	0.23
Total Processing Time (s)	6.23
Inference Time per Instance (ms)	4.69

**Table 3:** Stratified 5-fold cross-validation performance metrics of the deep neural network model.

Metric	Fold 1	Fold 2	Fold 3	Fold 4	Fold 5
Accuracy	0.9375	0.9167	0.9362	0.9362	0.9787
Balanced Accuracy	0.9375	0.9200	0.9375	0.9366	0.9792
Matthews CC	0.8758	0.8459	0.8798	0.8732	0.9583
Precision/PPV	0.9565	1.0000	1.0000	0.9565	1.0000
Sensitivity/Recall	0.9167	0.8400	0.8750	0.9167	0.9583
Specificity	0.9583	1.0000	1.0000	0.9565	1.0000
F1 Score	0.9362	0.9130	0.9333	0.9362	0.9787
NPV	0.9200	0.8519	0.8846	0.9167	0.9583
ROC AUC	0.9948	1.0000	0.9511	0.9583	0.9909

NPV: Negative predictive value; PPV: Positive predictive value.

**Table 4:** Stratified 5-fold cross-validation performance metrics of the support vector machine model.

Metric	Fold 1	Fold 2	Fold 3	Fold 4	Fold 5
Accuracy	0.7708	0.7500	0.8723	0.8298	0.8511
Balanced Accuracy	0.7708	0.7530	0.8714	0.8288	0.8496
Matthews CC	0.5538	0.5096	0.7468	0.6612	0.7070
Precision/PPV	0.7241	0.8095	0.8462	0.8077	0.8148
Sensitivity/Recall	0.8750	0.6800	0.9167	0.8750	0.9167
Specificity	0.6667	0.8261	0.8261	0.7826	0.7826
F1 Score	0.7925	0.7391	0.8800	0.8400	0.8627
NPV	0.8421	0.7037	0.9048	0.8571	0.9000
ROC AUC	0.8993	0.8504	0.9547	0.8913	0.9293

NPV: Negative predictive value; PPV: Positive predictive value.

**Table 5:** Stratified 5-fold cross-validation performance metrics of the logistic regression model.

Metric	Fold 1	Fold 2	Fold 3	Fold 4	Fold 5
Accuracy	0.7083	0.6250	0.8298	0.6809	0.7872
Balanced Accuracy	0.7083	0.6296	0.8297	0.6812	0.7880
Matthews CC	0.4226	0.2647	0.6594	0.3623	0.5771
Precision/PPV	0.6786	0.6842	0.8333	0.6957	0.8182
Sensitivity/Recall	0.7917	0.5200	0.8333	0.6667	0.7500
Specificity	0.6250	0.7391	0.8261	0.6957	0.8261
F1 Score	0.7308	0.5909	0.8333	0.6809	0.7826
NPV	0.7500	0.5862	0.8261	0.6667	0.7600
ROC AUC	0.7535	0.7409	0.8442	0.8134	0.8007

NPV: Negative predictive value; PPV: Positive predictive value.

**Table 6:** Stratified 5-fold cross-validation performance metrics of the K-Nearest Neighbors model.

Metric	Fold 1	Fold 2	Fold 3	Fold 4	Fold 5
Accuracy	0.7500	0.7917	0.8511	0.7021	0.6809
Balanced Accuracy	0.7500	0.7948	0.8505	0.7029	0.6803
Matthews CC	0.5071	0.5937	0.7024	0.4065	0.3612
Precision/PPV	0.7143	0.8571	0.8400	0.7273	0.6800
Sensitivity/Recall	0.8333	0.7200	0.8750	0.6667	0.7083
Specificity	0.6667	0.8696	0.8261	0.7391	0.6522
F1 Score	0.7692	0.7826	0.8571	0.6957	0.6939
NPV	0.8000	0.7407	0.8636	0.6800	0.6818
ROC AUC	0.8898	0.8583	0.8895	0.8025	0.8025

NPV: Negative predictive value; PPV: Positive predictive value.

**Table 7:** Stratified 5-fold cross-validation performance metrics of the AdaBoost model.

Metric	Fold 1	Fold 2	Fold 3	Fold 4	Fold 5
Accuracy	0.9792	0.8750	0.9149	0.9574	0.9149
Balanced Accuracy	0.9792	0.8748	0.9149	0.9565	0.9139
Matthews CC	0.9592	0.7496	0.8297	0.9180	0.8324
Precision/PPV	0.9600	0.8800	0.9167	0.9231	0.8846
Sensitivity/Recall	1.0000	0.8800	0.9167	1.0000	0.9583
Specificity	0.9583	0.8696	0.9130	0.9130	0.8696
F1 Score	0.9796	0.8800	0.9167	0.9600	0.9200
NPV	1.0000	0.8696	0.9130	1.0000	0.9524
ROC AUC	0.9983	0.9704	0.9221	0.9801	0.9837

NPV: Negative predictive value; PPV: Positive predictive value.

**Table 8:** Comparative performance summary of all models using stratified 5-fold cross-validation (mean and 95% confidence intervals).

Model	Accuracy	Balanced Accuracy	Matthews CC	Precision/PPV	Sensitivity/Recall	Specificity	F1 Score	NPV	ROC AUC
Deep Neural Network (DNN)	0.9414 (0.9247–0.9617)	0.9425 (0.9270–0.9623)	0.8872 (0.8581–0.9253)	0.9824 (0.9652–1.0000)	0.9022 (0.8693–0.9333)	0.9827 (0.9656–1.0000)	0.9399 (0.9223–0.9611)	0.9071 (0.8779–0.9359)	0.9701 (0.9620–0.9953)
Support Vector Machine (SVM)	0.8151 (0.7743–0.8553)	0.8150 (0.7753–0.8542)	0.6363 (0.5576–0.7126)	0.8000 (0.7604–0.8311)	0.8541 (0.7663–0.9083)	0.7759 (0.7217–0.8174)	0.8234 (0.7745–0.8640)	0.8426 (0.7706–0.8933)	0.9053 (0.8750–0.9335)
Logistic Regression	0.7267 (0.6640–0.7885)	0.7278 (0.6660–0.7888)	0.4582 (0.3353–0.5791)	0.7421 (0.6854–0.7997)	0.7137 (0.6120–0.8000)	0.7420 (0.6793–0.8087)	0.7245 (0.6472–0.7925)	0.7186 (0.6503–0.7844)	0.7906 (0.7579–0.8232)
K-Nearest Neighbors (KNN)	0.7541 (0.7032–0.8094)	0.7546 (0.7033–0.8099)	0.5120 (0.4085–0.6215)	0.7622 (0.7031–0.8243)	0.7604 (0.6940–0.8273)	0.7488 (0.6753–0.8203)	0.7588 (0.7097–0.8099)	0.7527 (0.6929–0.8142)	0.8482 (0.8137–0.8834)
AdaBoost	0.9289 (0.8989–0.9576)	0.9285 (0.8985–0.9572)	0.8590 (0.7982–0.9174)	0.9132 (0.8892–0.9375)	0.9519 (0.9103–0.9917)	0.9050 (0.8783–0.9315)	0.9318 (0.9027–0.9598)	0.9480 (0.9035–0.9905)	0.9713 (0.9453–0.9895)

NPV: Negative predictive value; PPV: Positive predictive value.

**Table 9:** Non-PD individual case study—CEM analysis results.

Variables	Original Value	PN Value	Change	PP Value	Interpretation
Age	−0.186	−0.189	→	−3.38 × 10^−9^	Minimal contribution
White Matter (WM)	−0.514	−0.514	→	9.82 × 10^−9^	Minimal contribution
Gray Matter (GM)	0.979	0.979	→	−2.13 × 10^−8^	Important: Strong positive contribution to “Normal” class
CSF	0.558	−0.649	↓↓	−1.18 × 10^−8^	Critical: Important decrease required for class change
Total Brain (BrainWM + GM)	0.630	1.378	↑↑	−1.73 × 10^−8^	Critical: Important increase required for class change
Intracranial Cavity (IC)	0.875	0.875	→	2.21 × 10^−8^	Minimal contribution
Cerebrum	0.562	0.562	→	2.97 × 10^−8^	Minimal contribution
Cerebellum	0.649	0.649	→	3.00 × 10^−9^	Minimal contribution
Brainstem	1.316	1.316	→	0.035	Important: Contribution to “Normal” class
Lateral Ventricle	−0.375	0.347	↑↑	−2.29 × 10^−10^	Critical: Largest change required feature
Caudate Nucleus	0.759	2.737	↑↑↑	−1.26 × 10^−8^	Critical: Very large increase for class change
Putamen	1.353	2.105	↑↑	−3.53 × 10^−8^	Critical: Important increase for class change
Thalamus	0.909	0.971	↑	−2.52 × 10^−8^	Moderate contribution
Globus Pallidus	−0.641	−0.641	→	2.62 × 10^−8^	Minimal contribution
Hippocampus	0.711	0.711	→	0.150	Important: Strong contribution to “Normal” class
Amygdala	−0.553	−0.553	→	−2.12 × 10^−8^	Minimal contribution
Nucleus Accumbens	0.423	0.845	↑↑	6.93 × 10^−9^	Important: Increase required for class change

**Table 10:** Parkinson’s patient case study—CEM analysis results.

Variables	Original Value	PN Value	Change	PP Value	Interpretation
Age	−0.186	−0.186	→	−3.38 × 10^−9^	Minimal contribution
White Matter (WM)	1.371	1.371	→	0.042	Important: Positive contribution to “Disease” class
Gray Matter (GM)	−0.794	−0.794	→	2.78 × 10^−8^	Important indicator in disease diagnosis
CSF	−0.126	0.463	↑↑	−0.039	Critical: Important increase required for class change
Total Brain (BrainWM + GM)	0.876	0.876	→	2.41 × 10^−9^	Minimal contribution
Intracranial Cavity (IC)	0.706	0.706	→	2.80 × 10^−8^	Minimal contribution
Cerebrum	0.921	0.921	→	−1.98 × 10^−8^	Minimal contribution
Cerebellum	0.209	0.209	→	−6.07 × 10^−9^	Minimal contribution
Brainstem	0.470	1.365	↑↑	−9.54 × 10^−9^	Critical: Important increase for class change
Lateral Ventricle	−0.375	−0.375	→	−2.29 × 10^−10^	Minimal contribution
Caudate Nucleus	−0.858	−1.110	↓	2.24 × 10^−9^	Decreased indicator in disease diagnosis
Putamen	−0.296	−0.296	→	7.79 × 10^−9^	Important indicator in disease diagnosis
Thalamus	0.292	0.292	→	0.083	Important: Contribution to “Disease” class
Globus Pallidus	−0.261	0.653	↑↑	−6.81 × 10^−9^	Critical: Important increase for class change
Hippocampus	−1.958	−1.958	→	−0.156	Critical: Strongest indicator of “Disease” class
Amygdala	−0.498	−0.498	→	−0.014	Important: Contribution to “Disease” class
Nucleus Accumbens	−0.670	−0.670	→	−0.023	Important: Contribution to “Disease” class

**Table 11:** Main comparative findings—non-PD individual vs. Parkinson’s patient.

Brain Region	Non-PD Individual (PP Value)	Parkinson’s Patient (PP Value)	Clinical Significance
Hippocampus	+0.150 (protective factor)	−0.156 (strongest disease indicator)	Most dramatic discriminative feature
Brainstem	+0.035 (contribution to normal diagnosis)	−9.54 × 10^−9^ (minimal)	Protective effect for normal diagnosis
White Matter (WM)	9.82 × 10^−9^ (minimal)	+0.042 (disease indicator)	Disease-specific change
Thalamus	−2.52 × 10^−8^ (minimal)	+0.083 (contribution to disease diagnosis)	Important role in disease process
Caudate Nucleus	Increase → disease risk	−0.858 (decrease → existing disease)	Opposite patterns: risk vs. existing disease
Putamen	Increase → disease risk	−0.296 (decrease → existing disease)	Indicator of basal ganglia atrophy
CSF	Decrease → disease risk	Increase → risk of return to normal diagnosis	Difference in compensatory mechanism
Amygdala	−2.12 × 10^−8^ (minimal)	−0.014 (disease contribution)	Limbic system involvement
Nucleus Accumbens	Increase → disease risk	−0.023 (disease contribution)	Change in reward system

**Table 12:** Group-wise comparison of brain volumetric features between PD patients and controls.

Variables	Control [Median (Min–Max)]	PD [Median (Min–Max)]	*p*-Value *
Age	58.00 (51.00–69.00)	58.00 (52.00–70.00)	0.276
White Matter (WM)	545.19 (13.59–1276.61)	618.33 (16.64–1221.45)	0.019
Gray Matter (GM)	650.78 (126.05–1325.84)	663.33 (55.74–1298.71)	0.06
Cerebro Spinal Fluid	200.76 (2.20–868.65)	194.16 (2.21–481.94)	0.709
Brain (WM + GM)	1273.15 (730.91–2276.11)	1322.65 (728.48–1799.69)	0.162
Intracranial Cavity	1460.38 (1064.54–3144.76)	1520.79 (909.82–2016.61)	0.187
Cerebrum	1120.45 (643.49–1948.99)	1168.45 (572.24–1597.53)	0.146
Cerebelum	134.98 (61.21–288.69)	136.02 (93.68–187.96)	0.549
Brainstem	25.34 (12.25–38.31)	21.50 (9.71–36.47)	<0.001
Lateral ventricles	0.44 (0.01–103.71)	0.19 (0.01–75.10)	<0.001
Caudate	10.12 (1.48–30.15)	12.04 (1.82–24.02)	<0.001
Putamen	6.42 (2.74–26.87)	6.12 (1.29–17.38)	0.08
Thalamus	10.52 (1.17–41.03)	11.64 (6.47–21.37)	0.091
Globus Pallidus	1.02 (0.02–6.43)	0.96 (0.25–2.36)	0.078
Hippocampus	6.61 (0.96–19.87)	6.35 (1.15–10.36)	0.21
Amygdala	0.31 (0.01–4.58)	0.12 (0.02–5.64)	0.001
Accumbens	0.25 (0.01–1.25)	0.11 (0.01–1.57)	<0.001

*: Mann–Whitney U test.

**Table 13:** Comparison of recent explainable AI studies for PD diagnosis and classification.

Reference	Dataset Type/Modality	ML Model(s) Used	XAI Method	Target/Task	Performance Results
Pang et al. (2021) [17]	Resting-state functional magnetic resonance imaging data	SVM	SHAP	Motor subtype classification of PD using multilevel rs-fMRI indices	SVM; AUC: 0.917
McFall et al. (2023) [14]	PD patients without dementia, recruited between 2003 and 2009 from the University of Alberta Movement Disorders Clinic	RF, LR, Gradient Boosting	TreeSHAP	Risk prediction of dementia progression in non-dementia PD patients	Random forest; AUC: 0.84
Chen et al. (2023) [16]	Diffusion tensor imaging data	DT, RF, XGBoost	SHAP	Automatic classification of PD patients with mild cognitive impairment using DTI-based features	XGBoost; accuracy: 91.67%
Junaid et al. (2023) [20]	Data extracted from the Parkinson’s Progression Markers Initiative	RF, LGBM, extra tree classifier, SVM, stochastic gradient descent	SHAP + LIME	Multi-class prediction of PD progression using multimodal time series data	LGBM; accuracy: 94.89%
Zhang et al. (2023) [13]	Data extracted from the Parkinson’s Progression Markers Initiative	Decision tree (DT), KNN, Naive Bayes, neural network, penalized LR, random forest, SVM, Extreme Gradient Boosting	SHAP	Risk prediction of PD based on multi-domain factors	Penalized logistic regression; AUC: 0.94
Ghaheri et al. (2024) [12]	Voice signals	Extreme Gradient Boosting (XGBoost), Light Gradient Boosting (LGBM), bagging, ensemble model	SHAP	Early-stage diagnosis of PD using acoustic biomarkers and feature importance interpretation	Ensemble method; accuracy: 85.42%
Tiwari et al. (2024) [19]	Data extracted from the Parkinson’s Progression Markers Initiative	AdaBoost, XGBoost, Gradient Boosting Classifier, DT, KNN, LR, Gaussian Naive Bayes (GNB)	LIME	Severity assessment of PD using clinical features and LIME explainability	AdaBoost; accuracy: 93.2%
CD et al. (2024) [18]	Voice signals	RF, DT, LR, KNN, SVM, Gradient Boosting, AdaBoost, XGBoost, CatBoost	SHAP + LIME	Diagnosis of PD using multiple ML models with SHAP and LIME explainability	XGBoost; accuracy: 94.8%
Ge et al. (2025) [15]	Transcranial ultrasonography + clinical	XGBoost	SHAP	Early diagnosis of PD using transcranial ultrasonography and clinical features	XGBoost; AUC: 0.81
Acikgoz et al. (2024) [41]	T2-weighted structural MRI	SE-ResNeXt with attention mechanism	—	Early diagnosis of PD using residual dense network	Accuracy: 94.44%, precision: 91.67%, sensitivity: 91.67%, specificity: 95.83%, F1 score: 91.67%, MCC: 87.50%
Welton et al. (2024) [42]	Midbrain MRI (SMWI, QSM, NMS)	Heuron IPD (DL model for N1 morphology), Heuron NI (DL model for N1 volume)	—	Diagnosis of PD using nigrosome-1 imaging features	AUC: 0.92 (N1 morphology), 0.90 (N1 volume), 0.94 (QSM-NMS composite marker), 0.98 (radiologist)
Li et al. (2024) [43]	Resting-state fMRI (PPMI dataset)	PD-ARnet (dual-branch 3D DL model)	—	Automated diagnosis of PD using ALFF and ReHo features	Accuracy: 91.6%, AUC: 92.8%, F1: 90.2%, precision: 94.7%, recall: 86.2%
Li et al. (2024) [44]	Structural MRI (582 images from 108 patients)	Improved YOLOv5 with CA, dynamic convolution, decoupling head	—	Detection and classification of Parkinson’s disease using enhanced deep learning model	Precision: 0.961, recall: 0.974, mAP: 0.986
Chang et al. (2025) [45]	Multimodal and multi-sequence PET/MR (CFT-PET, ADC-MRI)	ResNet18 (modified)	—	Classification of PD vs. MSA and normal controls	Best model (CFT-ADC): AUC = 0.96, accuracy = 0.97 (train); accuracy = 0.70 (test)
Alrawis et al. (2025) [46]	Structural MRI (PPMI, OASIS, MIRIAD)	FCN-PD (EfficientNet + Attention + FCN)	—	Diagnosis of PD using multi-dataset MRI data with hybrid deep architecture	Accuracy: 97.2% (PPMI), 95.6% (OASIS), 96.8% (MIRIAD)
Sangeetha et al. (2025) [47]	Structural MRI (axial, sagittal, coronal views)	ShCNN-Fuzzy-ZFNet, Deep Maxout Network, EfficientNet-B3 with attention	—	MRI-based PD classification using fuzzy convolutional hybrid model	Accuracy: 92.6%; TNR: 91.8%; TPR: 91.3%; NPV: 91.3%; PPV: 91.5%
Present Study	Volumetric structural MRI (brain morphometry)	SVM, LR, KNN, AdaBoost, Deep Neural Network (best)	Contrastive Explanation Method (CEM)	Diagnosis and feature-level interpretation of PD-related brain changes	Deep Neural Network; accuracy: 95.8%

## Data Availability

The data that support the findings of this study are available from the corresponding author upon reasonable request. Access to the dataset requires permission and can be obtained by contacting Fatma Ebru Algül (ebruycl86@yahoo.com) with appropriate justification for data use.

## Abbreviations

The following abbreviations are used in this manuscript:

PD	Parkinson’s Disease
CEM	Contrastive Explanation Method
XAI	Explainable Artificial Intelligence
MRI	Magnetic Resonance Imaging
DNN	Deep Neural Network
MLP	Multilayer Perceptron
WM	White Matter
GM	Gray Matter
CSF	Cerebrospinal Fluid
IC	Intracranial Cavity
PP	Pertinent Positive
PN	Pertinent Negative
ReLU	Rectified Linear Unit
ROC	Receiver Operating Characteristic
AUC	Area Under Curve
PPV	Positive Predictive Value
NPV	Negative Predictive Value
MCC	Matthews Correlation Coefficient
ANN	Artificial Neural Network
